# The impact of offering multiple cervical screening options to women whose screening was overdue in Dumfries and Galloway, Scotland

**DOI:** 10.1016/j.pmedr.2022.101947

**Published:** 2022-08-11

**Authors:** L. Wedisinghe, P. Sasieni, H. Currie, G. Baxter

**Affiliations:** a Department of Obstetrics and Gyanecology, Central Queensland Hospital and Health Service, Rockhampton, QLD 4700, Australia; b School of Cancer and Pharmaceutical Sciences, King’s College London, Great Maze Pond, London SE1 9RT, United Kingdom; c NHS Dumfries and Galloway, Dumfries and Galloway Royal Infirmary, Dumfries DG2 8RX, United Kingdom; d North Cumbria Integrated Care NHS Foundation Trust, Newtown Road, Carlisle CA2 7HY, United Kingdom

**Keywords:** Cervical cancer screening, Human papillomavirus, Screening overdue, Cervical screening uptake rate, National Health Service Scotland, Self-collection kit, Cervix unscreened women in Dumfries and Galloway, Vaginal self-sampling for HPV screening, Cervical smear testing non-responder, HPV, Human papillomavirus, CST, Cervical screening test, NHS, National Health Service, GP, General practice, PCR, Polymerase chain reaction, CIN, Cervical intraepithelial neoplasia, LBC, Liquid-based cytology, SS, Self-sampling

## Abstract

Most women who develop cancer have not been screened regularly. One in four women in Scotland, is overdue for cervical screening. Aim was to assess the impact of offering multiple cervical screening options to women whose screening is overdue.

A prospective cohort study including all women whose screening was overdue, aged 30–60 years in Dumfries and Galloway in 2012. Potentially eligible women (n = 4146) were identified split into six groups. Women aged 30–55 years were allocated to three different groups. Group 1 (letter, n = 1246), Group 2 (letter and kit, n = 221), Group 3 (letter, n = 2031). Women aged 56–60 years were allocated to: Group 4 (letter, n = 292), Group 5 (letter and kit, n = 292) and Group 6 (control, n = 64). Women who self-collected a vaginal sample were requested to complete a questionnaire. The percentages of women responding were 24 % (21–26), 32 % (25–38), 16 % (14–18), 15 % (11–20) and 12 % (9–17) in groups 1 to 5 respectively, compared with 3 % (0–11) among controls.

A significantly higher number of women (n = 383, 10 % of 3815) opted for self-sampling in comparison with undergoing a cervical screening test (CST) at the GP practice (n = 197, 5 %, x^2^ = 59.0, p < 0.0001). The Evalyn® Brush was well accepted (218/313 = 70 %) by those who requested self-sampling. Almost all (265/272 = 97 %) women who self-collected a vaginal sample said that if they had the option of self-sampling, they would regularly participate in future cervical screening.

Offering more flexible screening options, self-sampling in particular, appears to increase cervical screening participation.

## Highlights

• The study cohort was 4146 cervical screening non-responders in rural Scotland.
• They were offered multiple cervical screening options which includes self-sampling.
• A significantly more number of women preferred self-sampling to routine screening.
• Almost all women who self-collected said that if they had the self-sampling option.
• They will regularly participate in future cervical screening.

## 1 Introduction

Each day in the UK, around eight women are diagnosed with cervical cancer and two die from the disease. Most women who develop cancer have not been screened regularly. Concerningly, one in four women in Scotland, is overdue for cervical screening (Data and intelligence - Public Health Scotland, 2020).

Evidence suggests that practical barriers such as difficulty gaining access to a female smear-taker, communication issues, inaccessible locations, unfavourable appointment times, physical disability, previous bad experience, work and family commitments affect women’s decision-making more than attitudinal barriers (Waller et al., 2009, Marlow et al., 2015, Marlow et al., 2017). More flexible screening options such as home testing could overcome such barriers. Offering self-testing has increased screening participation among under- and unscreened women in many countries (Snijders et al., 2013, Verdoodt et al., 2015).

Several countries such as Australia and The Netherlands offer human papillomavirus (HPV) self-sampling to screening non-responders (Smith et al., 2016). Self-sampling is a powerful cervical screening tool for unscreened women. It is estimated that just one round of self-collected HPV screening at age 40 could avert 922 cancer diagnoses and 426 cancer deaths among unscreened women in Australia by age 84 (the number needed to treat for pre-cancer to avert each cancer diagnosis is 3.7) (Smith et al., 2016). The relative sensitivity of HPV testing of a self-collected vaginal sample is close to that for clinician-collected samples to detect CIN2+ (0.99; 0.97–1.02; (Arbyn et al., 2018) given a polymerase chain reaction (PCR) based assay is used. Since HPV testing of clinician samples has been shown to be more sensitive than cytology for detecting cervical pre-cancer, it follows that HPV testing of self-samples is more sensitive than liquid-based cytology (LBC) screening which was the standard in most screening programmes until very recently (Stanczuk et al., 2016). Women’s preference for self-sampling has not been evaluated among under- and unscreened women in Scotland, although it has been accepted by women in several settings. For example, the majority (77 %) of under- and unscreened women in an Australian study preferred self-sampling to clinician-based sampling in future screening (Polman et al., 2019). These data can be useful in shaping the future of national cervical screening programs.

Women in Scotland aged 20–60 years were eligible for cervical screening in 2012. They were offered a liquid-based cytology test every 3 years if they belong to ‘routine’ screening pathway. Women in the ‘non-routine’ screening pathway (e.g., due to previous unsatisfactory or a borderline change CST) were recalled for a repeat CST in 6 months. If a woman who belongs to a ‘routine’ (3 yearly screening recall) has not had a cervical cytology screening test in the previous 45 months was defined by the national screening program as a ‘non-responder’, as she has not been screened regularly for the past three and half years. Also, women whose CST was due sooner than 3 years (‘non-routine’ recall pathway) becomes a ‘non-responder’ at the 21st month since the last inadequate CST.

If a woman in a routine recall pathway did not respond to her screening ‘prompt’ letter for her CST which was due on month 36, she will be sent the first reminder at the 39th month and the second reminder at the 42nd month. She will be excluded from the recalling at the 45th month and considered a ‘non-responder’ for the next 27 months. No reminder will be sent during these 27 months. She will receive three more reminders (at 72, 75 and 81 months) and considered again as a ‘non-responder’ for another 27 months (from the month 81 to 108) during which time no reminder will be sent. This cycle (3 screening reminder letters in every 3 years) will continue until the woman has had a CST or become ineligible for screening. By contrast, a woman in ‘non-routine’ screening pathway will receive 4 reminder letters in every 3 years. For example, a woman who has had an ‘inadequate’ CST will receive her second cycle of reminder letters at months of 42, 44, 46 and 54 (since the last inadequate CST). ‘Non -responders’ from both of these pathways, aged between 30 and 60 years were the target population of this study. The aim of the study was to assess the impact of offering multiple screening options to the target population (women whose screening was overdue) and to determine the effect of different factors on screening uptake.

## 2 Methods

### 2.1 The study population and sampling

Dumfries and Galloway has a population of 148,000 people over a geographical area of 2,400 square miles, making it a rural population. The majority of the population was Caucasians. Around 36,500 women aged 20–60 are eligible for cervical screening. Of the eligible women, 76.6 % had been adequately screened in the previous 3.5 years and there were 6,109 cervical screening program non-responders (under- and unscreened women aged 20–60 years) (Data and intelligence - Public Health Scotland, 2020). In January 2012, there were 4,146 women aged 30–60 overdue cervical screening.

**Fig. 1:**
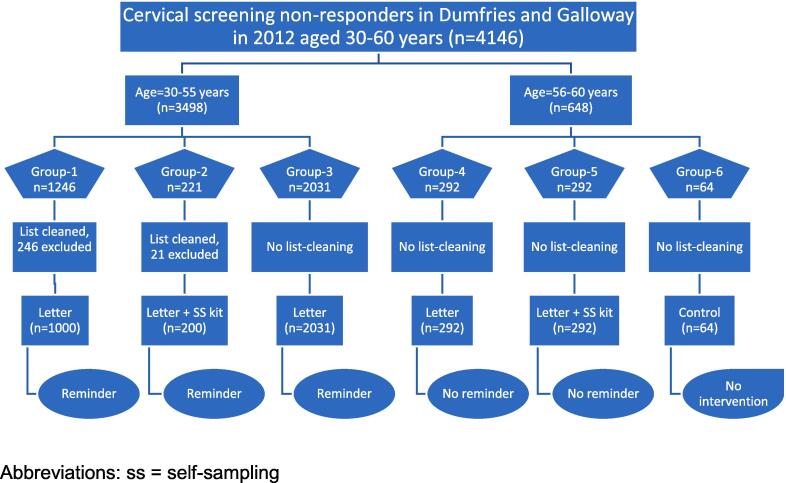
Different study groups with the recruitment flow chart. Abbreviations: ss = self-sampling.

The study population consisted of these 4146 ‘non responder’ women. It was divided into six groups based on age (30–55 or 56–60) and intervention (control, letter only, letter plus kit) (groups 1–6, Fig. 1). Group 1 (letter aged 30–55, n = 1246) and Group 2 (letter plus kit, aged 30–55, n = 221) A total of 246 women in Group 1 and 21 women in Group were excluded after database list–cleaning. Database list–cleaning was not done for any other group which was practically impossible. All remaining women aged 30–55 years were allocated to Group 3 (letter only, no list-cleaning, n = 2031). Women aged 56–60 years were allocated to three different groups: Group 4 (letter only, n = 292), Group 5 (letter plus kit, n = 292) and Group 6 (control, n = 64). Participants were recruited to 6 different groups sequentially over six months (from 15 March to 15 September 2012).

The non-responder database was ordered by the unique community health index (CHI) number. The 10-digit CHI number is based on the persons date of birth (DD/MM/YY) followed by 4 random digits that was generated by NHS database when the individual was first registered (at birth in most individuals). Thus, the database had a list of 4146 women which was arranged in a random order by the database software. Moreover, randomising this database once again was thought to be practically difficult and not cost-effective, considering the large numbers that is involved. Therefore, the Research and Development Support Unit decided to allocate women in order of the database into different study groups, 1 to 6. Proceeding in order of the database, the address and vital status of each woman aged 30–55 in the non-responder database was cross-checked against the hospital database (TOPAS Patient Administrative System) by the data manager of the Research & Development Support Unit. We continued cleaning the database until we had 1,000 valid live subjects with addresses that were the same in both databases. To do so, we examined 1,246 women to identify 1000 to invite. Five women were found to be deceased, two had left the country and 239 were excluded either because the addresses in the two databases did not match or because no address was found in the TOPAS database. Data cleaning continued until a further 200 live subjects with consistent addresses were identified: 21 women were excluded to identify these 200 women.

### 2.2 The intervention

**Fig. 2:**
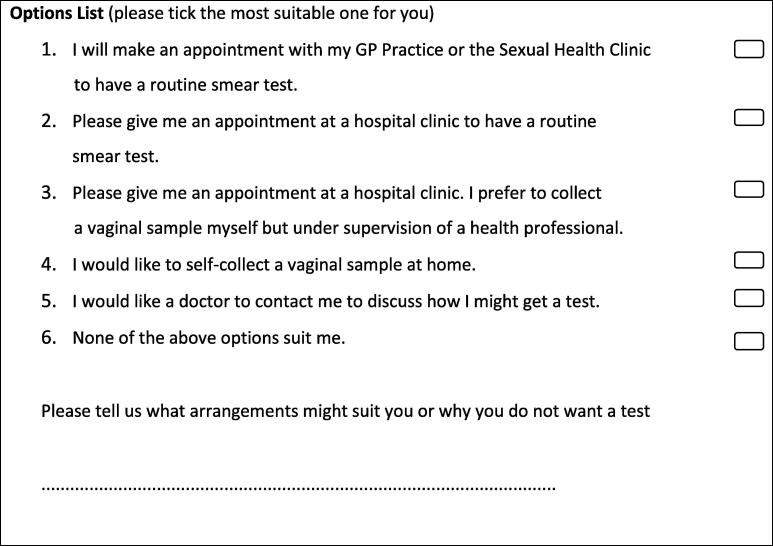
The list of cervical screening options offered.

All women in the five intervention groups were sent an initial letter inviting them to select one option from a list of six (Fig. 2). Women in Group 2 and Group 5 were sent a self-sampling kit along with the letter. Women in groups 1–3 who did not respond within two months were sent a reminder letter with the same options.

Women who requested home testing (option 4) in groups 1, 3 and 4 were sent a self-sampling kit (S1-4) whilst all eligible women in groups 2 and 5 were sent a kit with the letter. Women who asked for an appointment at a hospital clinic for an HPV test (option 3) were counselled at the clinic. They were offered two options for collection of a vaginal sample with the Evalyn® Brush following the manufacturer’s instructions (S3-4): self-collection under direct supervision of the clinician; or clinician collection. Every-one who opted for a hospital appointment chose to have a clinician to collect the sample. Women who requested a routine cervical screening test (CST) at a hospital clinic (option 2) were offered an appointment; those who attended had a speculum examination and a cervical sample taken by a clinician.

Women who tested HPV positive on their self-sample were encouraged to undergo a CST at the hospital clinic. All women who presented to the hospital’s CST clinic with a HPV positive self-sample were offered co-testing of the cervical sample: LBC with HPV testing - the clinician-collected cervical samples were first used to prepare an LBC slide and residual material was used for HPV testing. Women who were HPV positive, but LBC negative were invited to the annual follow-up clinic, where repeat CST, diagnostic colposcopy and/or punch biopsy were carried out after obtaining written consent. Each woman was strongly advised to accept their future National Health Service (NHS) cervical screening invitations regardless of the results of this study. All HPV tests used the Cervista HPV HR assay (Hologic). HPV results are not presented here.

A questionnaire was designed to assess the ease of use of the self-sampling kit (Supplement S2). This was sent along with the Evalyn® Brush to the women who wanted to self-collect. We requested that women complete the questionnaire after self-collecting the sample, and to return both the questionnaire and the sample in the pre-paid envelope.

### 2.3 Statistics

The study was designed to estimate the uptake of HPV self-sampling (Groups 1–5) and routine cervical screening (Group 6) with reasonable accuracy.

The reported self-sampling uptake rate in eight population-based, large-scale self-sampling studies ranged from 6.4 % (Szarewski et al., 2011)to 39.1 % (Sanner et al., 2009). The CST uptake rates in the self-sampling studies ranged from 4.1 % (Szarewski et al., 2011) to 17.6 % (Bais et al., 2007).

Assuming a 15 % HPV self-sampling uptake rate, the width of the 95 % confidence intervals (CI) based on 200 (Group 2), 300 (Groups 4 and 5), 1,000 (Group 1) and 2,000 (Group 3) invitation would be +/−5%, +/−4%, +/−2.5 % and +/−1.5 % respectively. Assuming 7 % uptake for CST, a sample of 64 in the control group would yield a 95 % CI of width +/-6%.

Binary logistic regression was used to analyse data using STATA (Version 12.0) software.

### 2.4 Ethical approval

The West of Scotland Research Ethics Service approved this study on 07/10/ 2011 (Reference No: 11/AL/0333).

## 3 Results

Letters were sent to 3815 potential participants in groups 1–5. A total of 775 (20 %) option lists were returned. The first option (to undergo routine screening at the GP practice) was selected by 197 participants (5 %), the second (to be screened at a hospital clinic) by 74 (2 %), the third (HPV testing at a hospital clinic) by 8 (0.2 %), the fourth (HPV testing of a self-sample) by 383 (10 %), the fifth (discuss with doctor) by 38 (1 %) and the sixth (opt out) by 81 (2 %). The total effective response rate (i.e., excluding option 6) was 18 % (701/3815). Around one in five women (267/1467 = 18 %) were excluded during the ‘list cleaning’. It was not possible to determine the number of women who had a CST at their GP practice in groups 1–5 because the actual screening uptake rate was unknown.

The number of women who chose these options varied between groups (Table 1). The percentages (95 % CI) of women responding were 24 % (21–26), 32 % (25–38), 16 % (14–18), 15 % (11–20) and 12 % (9–17) in groups 1 to 5 respectively, compared with 3 % (0–11) among controls. A significantly higher number of women (n = 383, 10 % of 3815) opted for HPV home testing (option 4) in comparison with undergoing a CST at the GP practice (option 2) (n = 197, 5 %, x^2^ = 59.0, p < 0.0001).

**Table 1:** Cumulated effective response at four months.

Group	1-Letter	%	2-Kit	%	3-Letter	%	4-Letter	%	5-Kit	%	6-None	%
Cohort	1246		221		2031		292		292		64	
Invited (%)	1000	80	200	90	2031	100	292	100	292	100	64	100
Mean age (SD)	43 (7.5)		43 (7.7)		43 (7.5)		58 (1.4)		58 (1.4)		58 (1.4)	
CST GP	63	6	8	4	108	5	15	5	6	2	2	3
CST Hospital	23	2	5	3	40	2	4	1	3	1	–	–
HPV Hospital	5	1	0	0	3	0	0	0	0	0	–	–
HPV Home	129	13	46	23	158	8	24	8	26	9	–	–
Discuss	16	2	4	2	16	0	1	0	1	0	–	–
Opted out	22	2	3	2	46	2	4	1	6	2	–	–
Total effective (95 % CI)	236	24 (21–26)	42	32 (25–38)	325	16 (14–18)	44	15 (11–20)	36	12 (9–17)	2	3 (0–11)

Abbreviations: CI = confidence intervals, CST = cervical screening test, GP = general practitioner, HPV = human papillomavirus testing of self-collected samples, SD = standard deviation.

Table 1: Cumulated effective response at four months.

A total of 313 self-sampling kits were sent to women who ordered one in groups 1, 3 and 4, and 492 kits were sent together with the letter to women in groups 2 and 5. A total of 279 samples were returned, of which five were excluded (one was lost in post, other was received after testing had ceased and 3 were collected in the hospital clinic). The numbers of vaginal samples available for analysis were 91 (9 %), 40 (20 %), 108 (5 %), 18 (6 %) and 25 (9 %) for groups 1–5, respectively.

### 3.1 Questionnaire analysis

All except one woman returned the completed questionnaire along with the sample. The remaining 272 questionnaires were analysed (Table 2).

**Table 2:** Analysis of 272 questionnaires.

**Question**	**Yes**	**%**	**No**	**%**	**NA**	**%**
Was the information **clear** enough to self-collect a sample?	264	97	4	1	4	1
Did you wish **more information**?	2	1	265	97	5	2
Was self-sampling **easy**?	265	97	4	1	3	1
Was self-sampling **uncomfortable**?	30	11	235	86	7	3
Was self-sampling **painful**?	12	4	256	94	4	1
Is self-sampling **acceptable** to you?	261	96	8	3	3	1
If you had the option of self-sampling, is it more likely that you would regularly participate in future cervical screening?	265	97	4	1	3	1
Please add any comments you may have below						

Abbreviations: NA = no answer.

Almost all participants who self-collected (265/272 = 97 %) indicated that the information provided was clear enough to self-collect a sample and they did not want any additional information. The same percentage found that self-sampling was easy (97 %) and acceptable (96 %). However, 11 % found self-sampling to be uncomfortable; it was painful for 4 %. A total of 265 women (97 %) said that if they had the option of self-sampling, they would regularly participate in future cervical screening.

Comments made in the free text box were categorised into types of reason for not responding to cervical screening: ‘practical, ‘attitudinal, ‘screening is not indicated’ or ‘unclear’ enough to put into one of the first three categories. Example comments (n = 26) and their classification are listed in S5.

Free comments were written by 25 % (68/272) of women who completed the questionnaire. The reason for not attending screening was unclear in 59 % (40/68) of the comments. Screening did not appear to be indicated for 3 % of the women who made free-text comments. When the reason for being a non-responder was clearly stated, it appeared to be a practical one for the majority (23/26 = 88 %).

## 4 Discussion

The most popular positive response was for self-collection (option 4; 10 %), followed by routine screening at the GP practice (option 1; 5 %), and screening at a hospital clinic (option 2; 2 %). HPV testing at a hospital clinic was selected by only eight women (option 3 = 0 %); therefore, this is not worth including in future research or service models. Participants in the self-sampling kit + reminder group (group 2) were 3.5 (2.4–5.1) times more likely to return a self-collected sample than the self-sampling letter + reminder groups (groups 1 and 3). This is consistent with *meta*-analysis which found that mailing kits to a woman’s home results in higher participation rates, but opt-in approaches did not (Arbyn et al., 2018).

Most women (70 %) who ordered the kit returned a sample. It was well accepted by those who did the self-test and highly rated and commended by women who self-collected a sample and returned it. Crucially, 97 % of those who returned a self-sample (and a questionnaire) said that they would regularly participate in future cervical screening, if they had the option of self-sampling. When the reason for not being screened in the past was clearly written by those who self-collected, it appeared to be a practical reason for the vast majority (88 %). A Swedish study (Darlin et al., 2013) collected data in a way that was similar to ours by sending a questionnaire to those who failed to respond one month after the first contact. The majority reasons for non-attendance were practical.

Self-sampling does not affect the detection of viral infections in comparison to clinician-collected samples (Gertler et al., 2021, Zander et al., 2021, Tsang et al., 2021). Self-sampling for HPV detection is not an exception (Snijders et al., 2013). The importance of self-sampling for HPV detection was highlighted during the coronavirus pandemic (Lozar et al., 2021, Adame et al., 2019, Canfell et al., 2021).

### 4.1 Strengths

This study included the whole target population (all cervical screening non-responders over the age of 30). A wide range of potential reasons for non-participation in organised (NHS) cervical screening were explored in this study.

### 4.2 Limitations

Six study groups were recruited sequentially over 6 months (due to large number of participants involved) so that there could be seasonal effects and age effects that might confound the uptake in different groups. List-cleaning was carried out only in groups 1 and 2 which is a limitation of this study.

The control group did not include women aged 30–55; therefore, it is not representative of the whole population, and the relative effect on total screening uptake could not be measured. It included 10 % (64/648) of women from the second database that we received of non-responders aged 56–60. Moreover, relatively small sample sizes in groups 2, 4 and 5 may confound results. The CST uptake rate in this cohort of 64 women without any intervention was 3 % over four months. This is comparable with statistics from London (Szarewski et al., 2011), which were 4.5 % over six months with one reminder letter.

## 5 Conclusion

It is known that the relative sensitivity of HPV screening of self-collected vaginal samples is similar to that of clinician-collected cervical samples in detecting cervical pre-cancer or cancer (Arbyn et al., 2018) given a polymerase chain reaction (PCR) based assay is used. Offering self-sampling to women whose screening is overdue appears to increase cervical screening participation. Almost all (97 %) women who self-collected a vaginal sample said that if they had the option of self-sampling, they would regularly participate in future cervical screening. Therefore, we recommend that the option of self-sampling be included in cervical screening programs for which the primary screening strategy is HPV detection.

## Funding

Rovers Medical Devices B. V. Netherlands sponsored Evalyn self sampling devices while NHS Dumfries and Galloway sponsored other main expenses, including the costs of self-sampling kits and postage. Hologic UK ltd provided HPV testing kits and other consumables. They together with the Scottish HPV Reference Centre provided some intellectual support. The principle investigator (main author) is responsible for some minor expenses. The Research Department of the Central Queensland Hospital and Health Service paid the APC.

## CRediT authorship contribution statement

**L. Wedisinghe:** Conceptualization, Methodology, Writing – review & editing. **P. Sasieni:** Supervision. **H. Currie:** Supervision. **G. Baxter:** Supervision.

## Declaration of Competing Interest

The authors declare that they have no known competing financial interests or personal relationships that could have appeared to influence the work reported in this paper.

## Data Availability

Data will be made available on request.
