# Supplementary material for: The impact of offering multiple cervical screening options to women whose screening was overdue in Dumfries and Galloway, Scotland
Source: Prev Med Rep. 2022 Aug 11;29:101947. doi: 10.1016/j.pmedr.2022.101947 (PMC9502330; doi:10.1016/j.pmedr.2022.101947)
Supplement: Supplementary data 1 [file mmc1.docx]

**Supplementary Material**

**Figure S1:** Self-sampling kit pack- a list of contents that was sent out to women

| 1. The sealed, sterile Evalyn device which is placed inside a transparent, ‘peel and seal’ type plastic bag which measures (10x25cm). A white colour, absorbent pad (12x9cm) is also seen inside the plastic bag (packed according to the UN3373 standards packaging instruction P650). 2. A pre-paid, jiffy bag (170x225cm) with the return address 3. Evalyn brush patient information leaflet 4. Questionnire-1 with a check list 5. Two copies of the consent form 6. The study’s patient information leaflet 7. A list of frequently asked questions 8. A letter addressed to the participant |
| --- |

**Figure S2:** Self-sampling kit pack- a photo of self-collected sample that was returned in pre- paid post


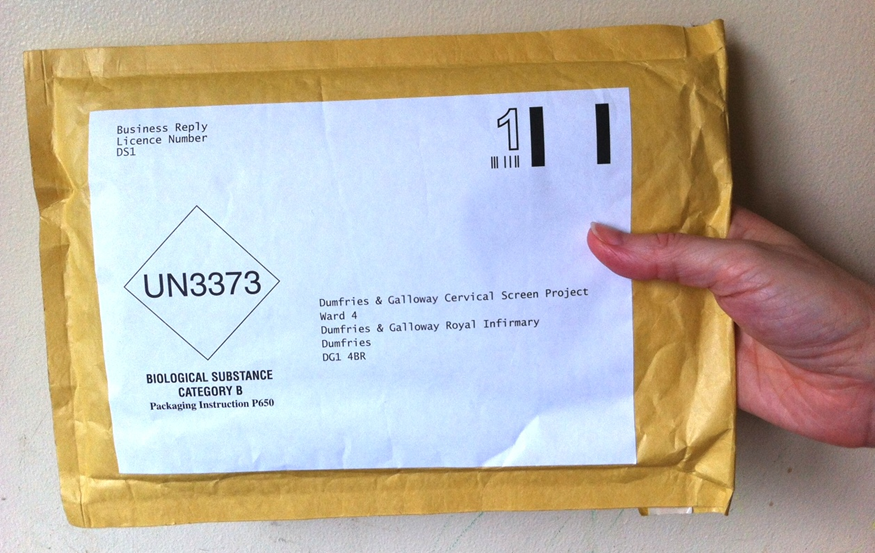


**Figure S3:** A summary of instructions for vaginal self-collection

| The woman was advised to read the Evalyn brush patient information leaflet and our check list carefully, before the sample was collected. It is a two-page leaflet with colorful illustrations.  The information leaflet instructs the female to spread the labia with one hand and insert the Evalyn brush into the vagina with the other, until the ‘wings’ of the Evalyn device come to contact with the labia. Then she is instructed to push the plunger (handle) in, hold it firmly and rotate the plunger 5 times clockwise, remove it and pull the plunger back, put the cap on and place it back in its plastic housing. This should be put in a transparent plastic bag which should be sealed after peeling its sticker off.  This plastic bag and the signed consent form and completed questionnaire were dispatched to Dumfries Laboratory in the pre-paid, first class mail jiffy bag provided. The woman was asked to put the large size envelope in a post box. |
| --- |

**Figure S4:** Evalyn brush user information leaflet


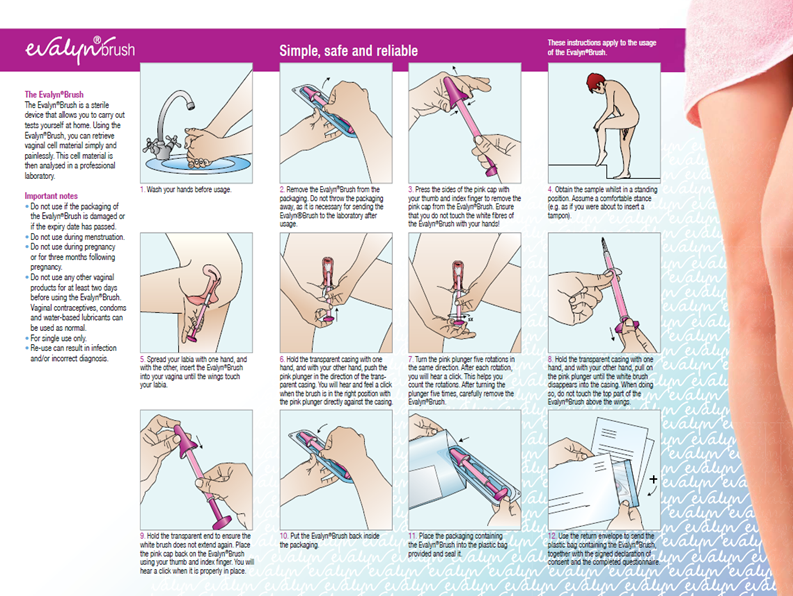


**Figure S5:** Cervical screening barriers- free comments written in questionnaires by

Participants

1. practical reasons

| 1. “I suffer from IBS, so I don’t like to have people prodding at me at times when it flares up”. (43 years old, last CST 7 years ago) 2. “I suffer from agoraphobia, so it makes it difficult to get to surgery”. (42 years old, last CST 8 years ago) 3. “I suffer from a very unpleasant vaginal discharge, too embarrassed to allow a doctor to perform a smear”. (45 years old, last CST 5 years ago) 4. “I have some memory problems”. (56 years old, last CST 5 years ago) 5. “I have very painful hips & this form of screening was much more comfortable”. (58 years old, last CST 6 years ago) 6. “I personally know my GP and the practise nurse I have had a bad smear experience at my practice”. (39 years old, last CST 4 years ago) 7. “I have been sexually abused”. (36 years old, last CST 10 years ago) 8. “Self-sampling may be the only way transgender persons will have screening”. (38 years old, last CST 8 years ago) 9. “Fibroids obstructing the cervix - taking a smear was unsuccessful in 4 different days”. (53 years old, last CST 4 years ago) 10. “I find it extremely painful to have a normal smear test and this was painless, easy & quick.”. (43 years old, last CST 4 years ago) 11. “Previously the nurse had a problem due to the position of the cervix- is this sample adequate?”. (48 years old, last CST 6 years ago) 12. “This way of screening is much more private and so much easier to fit into your life”. (41 years old, last CST 4 years ago) 13. “Nice to be able to do it at home when you are free and relaxed. Thank you”. (44 years old, last CST 11 years ago) |
| --- |

1. attitudinal reasons

| 1. “I have made it to 32 years old without having a smear- ridiculously avoided because of embarrassment. If this self-sampling was an option, I would participate and get tested regularly”. (32 years old, never had a CST) |
| --- |

1. screening was not indicated

| 1. “Declined last smear invite as I work in NHS England ad for me invite would be given not 3 years as in NHS Scotland”. (36 years, had a CST 2 years ago in England but never had one in Scotland) 2. “This was easy to do, as I never had one before as I am not sexually active, and I have not had sex yet”. (31 years, never had a CST) |
| --- |

1. the reason is not clear

| 1. “I would do this every year rather than go to doctor, even though my doc is great!” (36 years old, last CST 12 years ago) 2. “No lasting aftereffects but didn’t expect spotting.” (54 years old, last CST 5 years ago) 3. “Slightly uncomfortable, noted small spot of blood after taking sample.” (56 years old, last CST 6 years ago) 4. “I was sore on insertion, but the test was easy and once inside was OK” (57 years old, last CST 4 years ago) 5. “I don’t think I should have to take part in a study to have this option.” (56 years old, last CST 9 years ago) 6. “So much paper to work through may put people off participating in this study.” (55 years old, last CST 6 years ago) 7. “Needed translation” (37 years old Eastern European, never had a smear) 8. “I did struggle with trying to work out how to seal the bag!” (46 years old, last CST 7 years ago) 9. “It would have been better (for the kit) to come in a Jiffy bag.” (60 years old, last CST 9 years ago) 10. “No mention about the absorbent paper in the instruction leaflet.” (this has been removed by the participant, 55 years old, last CST 6 years ago) |
| --- |
